# On the improvement of reinforcement active learning with the involvement of cross entropy to address one-shot learning problem

**DOI:** 10.1371/journal.pone.0217408

**Published:** 2019-06-19

**Authors:** Honglan Huang, Jincai Huang, Yanghe Feng, Jiarui Zhang, Zhong Liu, Qi Wang, Li Chen

**Affiliations:** 1 College of Systems Engineering, National University of Defense Technology, Changsha, Hunan, China; 2 College of Aerospace Science and Engineering, National University of Defense Technology, Changsha, Hunan, China; Wroclaw University of Science and Technology, POLAND

## Abstract

As a promising research direction in recent decades, active learning allows an oracle to assign labels to typical examples for performance improvement in learning systems. Existing works mainly focus on designing criteria for screening examples of high value to be labeled in a handcrafted manner. Instead of manually developing strategies of querying the user to access labels for the desired examples, we utilized the reinforcement learning algorithm parameterized with the neural network to automatically explore query strategies in active learning when addressing stream-based one-shot classification problems. With the involvement of cross-entropy in the loss function of Q-learning, an efficient policy to decide when and where to predict or query an instance is learned through the developed framework. Compared with a former influential work, the advantages of our method are demonstrated experimentally with two image classification tasks, and it exhibited better performance, quick convergence, relatively good stability and fewer requests for labels.

## Introduction

In recent decades, machine learning has attracted increasing attention from both industry and academia and shown its great power in universal applications, such as pattern analysis [1], knowledge discovery and discipline prediction. As acknowledged in this domain, data resources are crucial in learning tasks. A direct strategy to process data and incorporate human experience is to formulate labels for examples. In small-scale datasets, precise annotation based on expert knowledge is acceptable. However, when large-scale datasets are used for complicated tasks, complete and perfect annotations are no longer viable, due to the reality that labeling process for these datasets is labor-intensive, costly in terms of time and money, and dependent on domain experience. With the increase of dataset volume, the learning system tends to generalize better, but the cost of annotation dramatically increases [2]. Meanwhile, former studies have revealed that obtaining the ground truth label of a dataset not only requires the participation of a large number of experts in the field, but also takes more than 10 times longer to label the instance as to collect it [3]. In contrast, accessing a massive number of unlabeled instances is relatively easy. The availability of a massive number of unlabeled examples as well as the potential task-beneficial information buried in them has led to enlightenment through some effective paradigms employed in the learning domain, including semi-supervised learning and active learning. The goals of these emerging paradigms are to take advantage of the unlabeled datasets for performance promotion and to reduce workloads of human experts. Semi-supervised learning has developed quickly in recent years, exploiting statistical or geometrical information in unlabeled examples to enhance the generalization. Notably, however, the involvement of the unlabeled examples in a semi-supervised framework may be inappropriate and degrade the original accuracy in certain scenarios. Another powerful learning paradigm-active learning is significantly distinct from semi-supervised learning in theory and practice. The difference is that the active learning algorithm simulates the human learning process to some extent: selects part of instances to label and join the training set, and iteratively improves the generalization performance of the classifier. Therefore, this algorithm has been widely used in information retrieval [4], image and speech recognition [5–11], and text analysis [12–14] in recent years.

The core of traditional active learning methods is to formulate criteria for selecting samples, and commonly-used methods include uncertainty sampling [15], query-by-committee [16], margin [17], and representative and diversity-based sampling [18]. However, determining which approach is better is difficult since each approach starts from a reasonable, meaningful, and completely different motivation. To the best of our knowledge, no universal method that performs best on all datasets currently exists. These limitations drive us to explore new frameworks to address the sample-selecting problem. Observing that human beings can learn new concepts from a single example [19], we sought to design an artificial intelligence agent that can inherit a similar capability and pose fewer requests for labeling new examples during the training process [20]. An ideal case in active learning is one in which labeling of critical examples is still required, but the frequency can be minimized. We preferred a model that learns active learning algorithms via reinforcement learning [21, 22], rather than a hand-design criterion. More specifically, the selection or design of a new example labeling strategy can be performed automatically.

Therefore, we propose a novel learning method, that can not only learn to classify instances with little supervision but also capture a relatively optimal label query strategy as well. Our method is mostly inspired by the work of Mark Woodward et al. [23] and can be viewed as a practical extension of that work. Our model falls into the class of stream-based active learners, which is based on the online setting of active learning. The use of reinforcement learning by an active learner to solve a continuous decision problem is a natural fit since each query action affects the next decision (when and which instance to query based on the state of the basic learner). Accordingly, the active query system trained by the reinforcement learning can learn a cogent, non-myopic strategy [24], and make effective decisions with little supervision.

Our primary contribution in this work is improvement of the influential active one-shot learning (AOL) model introduced by Mark Woodward et al. [23]. Woodward’s work is known to be the first practice of reinforcement learning with deep recurrent models in the task of active learning. With additional metric cross entropy involved in the loss function of Q-learning, we significantly accelerate the convergence speed, avoid the gradient vanishing problem, improved the stability, reduce the number of requested labels, and improve the level of accuracy in comparison with the former work of Mark Woodward et al. [23]. Meanwhile, we evaluate the model on Omniglot [19, 25, 26](“active” variants of existing one-shot learning tasks [27]), and the experimental results show the efficiency of our model in exploring label querying strategies. We empirically demonstrate that our model can achieve better performance with fewer iterations and learn a query strategy based on uncertainty [28] of instances in an end-to-end fashion. Accordingly, the workload of human experts can be partially reduced during the learning process.

## Related work

The setting of active learning is mainly based on three scenarios: (i) membership query synthesis, (ii) pool-based sampling, and (iii) stream-based selective sampling [29]. In the membership query synthesis scenario, the learner can select a new instance to label from the input space, or it can generate a new instance. In the pool-based scenario, the learner can request labels for any instance from a large amount of historical data. Finally, in the stream-based active learning scenario, instances can be continually obtained from the data stream and presented in an exogenously-determined order. The learner must instantly decide whether to request a label for the new instance [30]. Various practical scenarios have benefited from the idea of active learning, including movie recommendation [31–33], medical image classification [34], natural language processing.

In recent years, reinforcement learning has gained considerable attention. Due to its capability of interacting with the environment and providing a good approximation of the objective value based on relevant feedback, this method is theoretically suitable for online, real-time forecasting and decision-making. Particularly for specific complex tasks, in the unknown environment, reinforcement learning can learn the optimal strategy by exploration and exploitation. This learning framework has also been successfully applied to solve complex predictive and control problems in virtual environments [21].

In this article, we mainly consider the setting of the third scenario, single pass stream-based online active learning. Many studies have focused on active learning based on data streams [35–37], and a common opinion is that the choice of a proper instance to label should be based on maximizing the expected informativeness of the labeled instances [30]. In general, most of these methods rely strongly on heuristics, such as similarity measures between former instances and current instances [38] or the extent of uncertainty in label prediction [36, 38, 39]. To move away from engineered selection heuristics, we introduce a model learning active learning algorithm end-to-end via reinforcement learning. The premise of active learning is that costs associated with requesting labels and making false predictions exist [23]. Reinforcement learning can optimize these costs by explicitly setting them and directly identifying an action strategy. Therefore, we believe that combining reinforcement learning with active learning is a reasonable and appealing approach. Some recent studies have been based on a similar inspiration. Woodward and Finn [23] first applied reinforcement learning with deep recurrent models to the task of active learning. Bachman et al. [27] and Pang et al. [24] investigated a pool-based active learning algorithm via meta-learning. The same idea emerged in the artificial intelligence classification systems developed by Puzanov and Cohen [20]. Recent approaches, such as meta-learning and one-shot learning, are closely related to our model. Santoro et al. [25] proposed a supervised learning model using meta-learning with memory-augmented neural networks, which approached the same task as ours. The practical applications of these methods show that they are good solutions to the cold start problem [31, 40–42]. In our work, a deep recurrent neural network [43] function approximator is used to represent the action-value function and the cross entropy [44] term is introduced to the loss function to improve the performance of the algorithm.

## Model description

In this section, we present a novel model based on the reinforcement one-shot active learning (ROAL) framework, which can monitor a stream of instances and select an appropriate action (classify or query the label) for each arrival instance. Our model metalearns a query strategy, which intelligently captures the time and population of instances that are worth to query. In present study, a long short-term memory (LSTM), which is connected to a linear output layer, is used to approximate the action-value function.

### Task description

**Fig 1:**
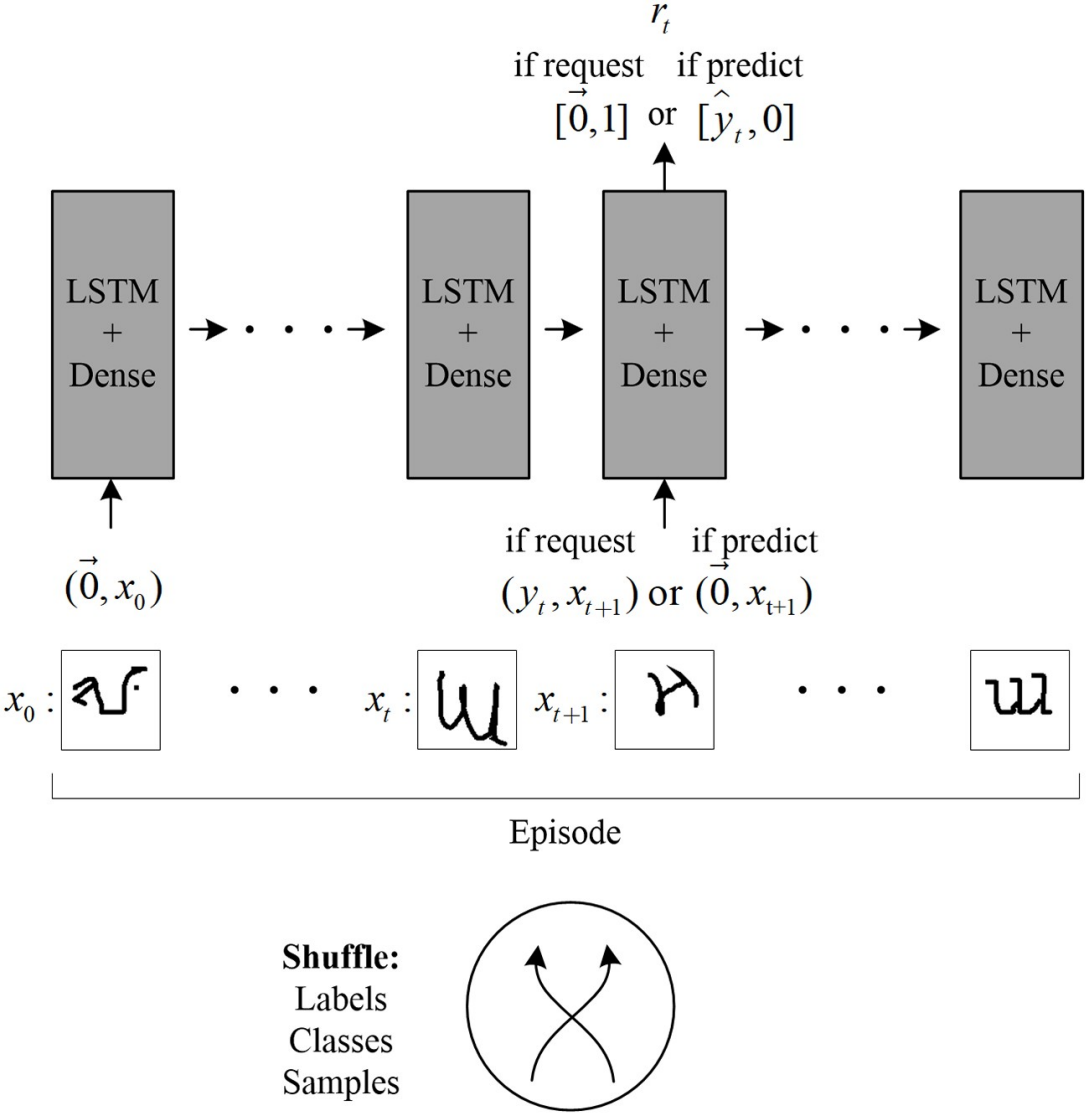
Task structure diagram. For images in the dataset, the classes and their labels and the specific samples are shuffled and randomly presented at each episode. At each time step, the input of the model is an image along with a vector which depends on the output of the previous instance. The output of the model is a one-hot vector of length *k* + 1, where *k* is the number of classes per episode. If the model requests the label of *x*_*t*_, it sets the final bit of the output vector to 1. Thus, the reward for this label request action is *R*_*req*_. The true label *y*_*t*_ of image *x*_*t*_ is then provided at the next time step along with the next image *x*_*t*+1_. Alternatively, if the model makes a prediction of *x*_*t*_, it sets one of the first *k* bits of the output vector, representing $\hat{y}$. The reward for this action is *R*_*cor*_ if the prediction is correct or *R*_*inc*_ if not. If a prediction is made at time step *t*, then no information regarding its true label *y*_*t*_ is supplied at the next time step *t* +1.

In the stream-based online active learning scenario, obtaining the ground truth label of a data instance is costly; therefore, an algorithm is required to judiciously determine the population of instances to label [29, 45]. In this setting [29, 46], the algorithm takes an action and chooses whether or not to request the ground truth at the time that the instance arrives. The classification task that we focus on is a stream of images, in which a decision must be made to either query or predict the label. Similar to works on one-shot learning [25, 26], the behavior of our model is refined over short training episodes and a small amount of examples per class to maximize the performance of the test episodes for instances that are not encountered in training. The structure of our active learning task is shown in Fig 1. At each time step of the episode, the model receives an instance *x*_*t*_, and need to decide to execute an action. Assume that in each episode, up to *M* possible classes exist. Let *a*_*t*_ be the action at time step *t*; then, the action space is defined as follows:

$$A≜\{c_{1},\ldots ,c_{M},a_{req}\}$$ (1)

Action *a*_*t*_ = *c*_*i*_ is taken when the model classifies the instances under category *i* without requiring the true label at time *t*. Action *a*_*t*_ = *a*_*req*_ is taken when the model requests the true label *y*. Here we set the action *a*_*t*_ as a one-hot vector consisting of the optionally predicted label $\hat{y}$ that is followed by a bit for requesting the label. The model can either make a label prediction or request the label since only one bit can be 1. If the model requests the label of instance *x*_*t*_, then no prediction will be made, and the true label *y*_*t*_ of the instance will be sent into the model at the next observation *o*_*t*+1_ along with a new instance *x*_*t*+1_. If the model decides to predict, then no request will be made and a $\vec{0}$ will be included in the next observation instead of the true label.

*r*_*t*_ is the reward received after action *a*_*t*_ in state *s*_*t*_, and *γ* represents the discount factor for future rewards. At each time step, one of three rewards is given depending on the chosen action: *R*_*cor*_ for correctly predicting the label, *R*_*inc*_ for incorrectly predicting the label, or *R*_*req*_ for requesting the label. The aim is to maximize the sum of the rewards received in this episode.

$$r_{t}=\left\{ \begin{array}{c} R_{cor},\;\text{if}\;\text{predicting}\;\text{and}\;{\hat{y}}_{t}=y_{t}\;\\ R_{inc},\;\text{if}\;\text{predicting}\;\text{and}\;{\hat{y}}_{t}y_{t}\\ R_{req},\;\text{if}\;\text{a}\;\text{label}\;\text{is}\;\text{requested} \end{array}\right.$$ (2)

### Methodology

**Fig 2:**
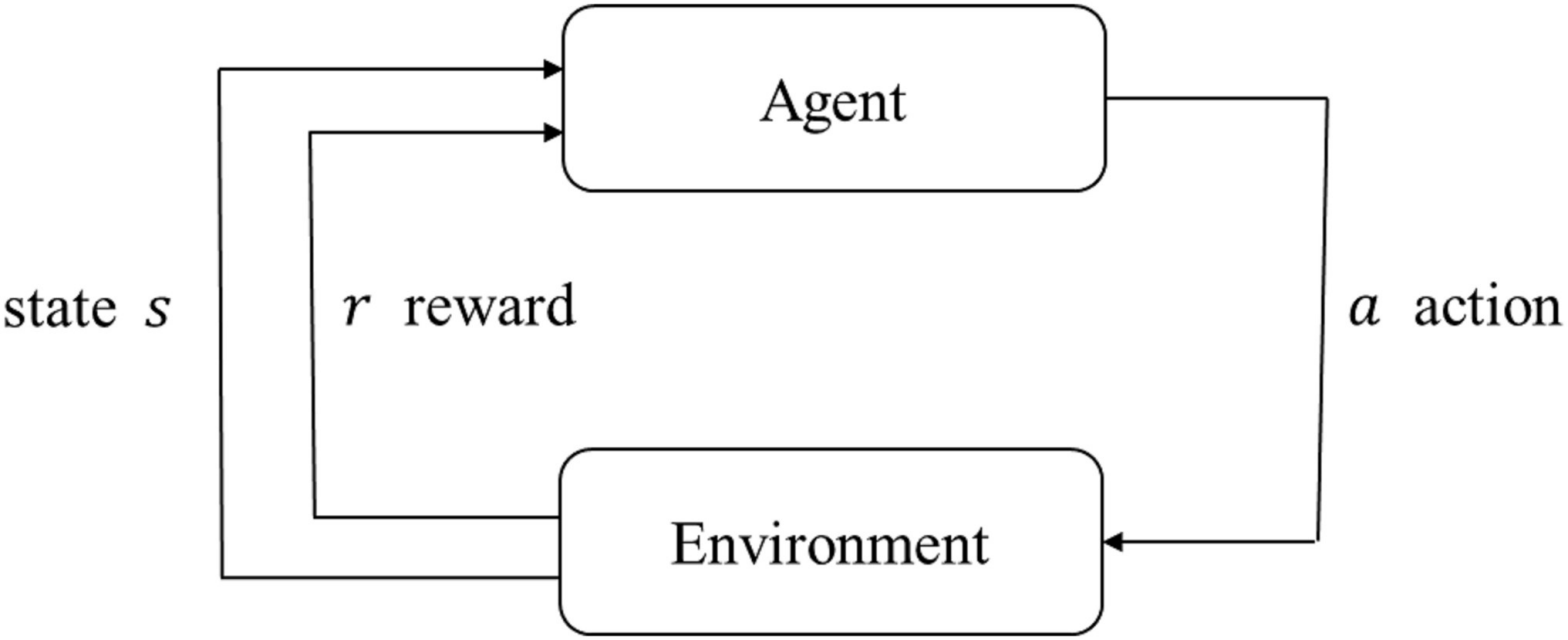
Basic reinforcement learning model. When the Agent performs an action, the state of the environment is changed, and a reward signal is feedback to the Agent. The Agent selects the next action according to the reward signal and the current state of the environment, and the selection principle is to increase the probability of receiving positive reinforcement (maximizing rewards). The actions selected affect not only the immediate rewards, but also the state of the environment at one point and the final values.

Reinforcement learning aims at seeking practical and superior strategies in complicated control and prediction tasks by interacting with environment. Through explorations as well as exploitations, it can estimate the goodness of a policy and perform improvements based on experience information. The basic structure of reinforcement learning can be seen in Fig 2. And an efficient model-free reinforcement learning method Q-learning is employed in this paper to learn an optimal strategy that can maximize the expected sum of discounted future rewards. Q-learning has been widely used in a variety of decision-making problems [47], mainly because it can estimate the expected utility from the available operations and adapt to stochastic transitions without prior knowledge of the system model [48].

Reinforcement learning requires a definition of an objective function to show the benefit of an action in the long run. The idea of Q-learning is not to estimate the environmental model, but to optimize a Q function that can be directly calculated. The Q function reflects the gain obtained after performing action *a*_*t*_ under state *s*_*t*_, and then accumulates the reinforcement value according to the discount of the best action sequence performed later:

$$Q\left(s_{t},a_{t}\right)=r_{t}+\gamma \;{\text{max}}_{a_{t+1}\in A}Q\left(s_{t+1},a_{t+1}\right)$$ (3)

Here let *π*(*s*_*t*_) be a policy which is taken at *s*_*t*_, and outputs an action *a*_*t*_ at time *t*. A policy that is better than or equal to other policies always exists, and this policy is called the optimal policy π*(*s*_*t*_). The optimal policy is the strategy that maximizes the optimal action-value function Q*(*s*_*t*_, *a*_*t*_). In other words, the action that the model selects is given by the optimal policy π* which is calculated by maximizing the optimal action-value function *Q*(*s_t_*, *a_t_*)

$$a_{t}=\pi^{*}(s_{t})={\text{argmax}}_{a_{t+1}\in A}Q^{*}(s_{t},a_{t})$$ (4)

According to Bellman equation, the optimal action-value function can be derived as follows:

$$Q^{*}(s_{t},a_{t})=E_{s_{t+1}}[r_{t}+\gamma \;{\text{max}}_{a_{t+1}\in A}Q^{*}(s_{t+1},a_{t+1})]$$ (5)

Normally, a function approximator is used to represent *Q*(*s*_*t*_, *a*_*t*_), and its parameters are optimized by minimizing the Bellman error. Woodward et al. [23] derived the loss function as follows:

$$L(\theta )≔\sum_{t}{\left[Q(o_{t},a_{t})-(r_{t}+\gamma \;{\text{max}}_{a_{t+1}\in A}Q^{*}(s_{t+1},a_{t+1}))\right]}^{2}$$ (6)

Where *θ* represents the parameters of the function approximator, and *o*_*t*_ represents the observations, such as images, that the agent receives.

However, in the early stages of training, this loss function tends to be inefficient and prone to encounter the gradient vanishing phenomenon, because the loss function here only considers the maximum value of *Q*. In order to avoid these shortcomings and accelerate the training to advance the efficiency of our model, we introduce the cross-entropy of *Q* values and labels in the loss function. Cross-entropy is an important concept in Shannon’s information theory that is mainly used to measure the difference information between two probability distributions. The intuition is that we want to increase the similarity of the label prediction probability distribution output by the model to the probability distribution of the real label. This method has been applied in many fields of machine learning. Inspired by this idea, we design our loss function as follows:

$$L(\theta )≔\left\{ \begin{array}{c} \sum_{t}(\begin{array}{c} {\left[Q_{\theta }(o_{t},a_{t})-(r_{t}+\gamma \;{\text{max}}_{a_{t+1}\in A}Q^{*}(s_{t+1},a_{t+1}))\right]}^{2}\;\\ -p(Q(o_{t},a_{t}))\text{log}(q(label(t)) \end{array})\;\text{if}\;\text{predicting}\;\\ \sum_{t}{\left[Q(o_{t},a_{t})-(r_{t}+\gamma \;{\text{max}}_{a_{t+1}\in A}Q^{*}(s_{t+1},a_{t+1}))\right]}^{2}\;\text{if}\;\text{a}\;\text{label}\;\text{is}\;\text{required} \end{array}\right.$$ (7)

Where *p*(*Q*(*o*_*t*_, *a*_*t*_)) represents the probability distribution of *Q*(*o*_*t*_, *a*_*t*_) and *q*(*label*(*t*)) represents the probability distribution of the true label at time step *t*.

**Fig 3:**
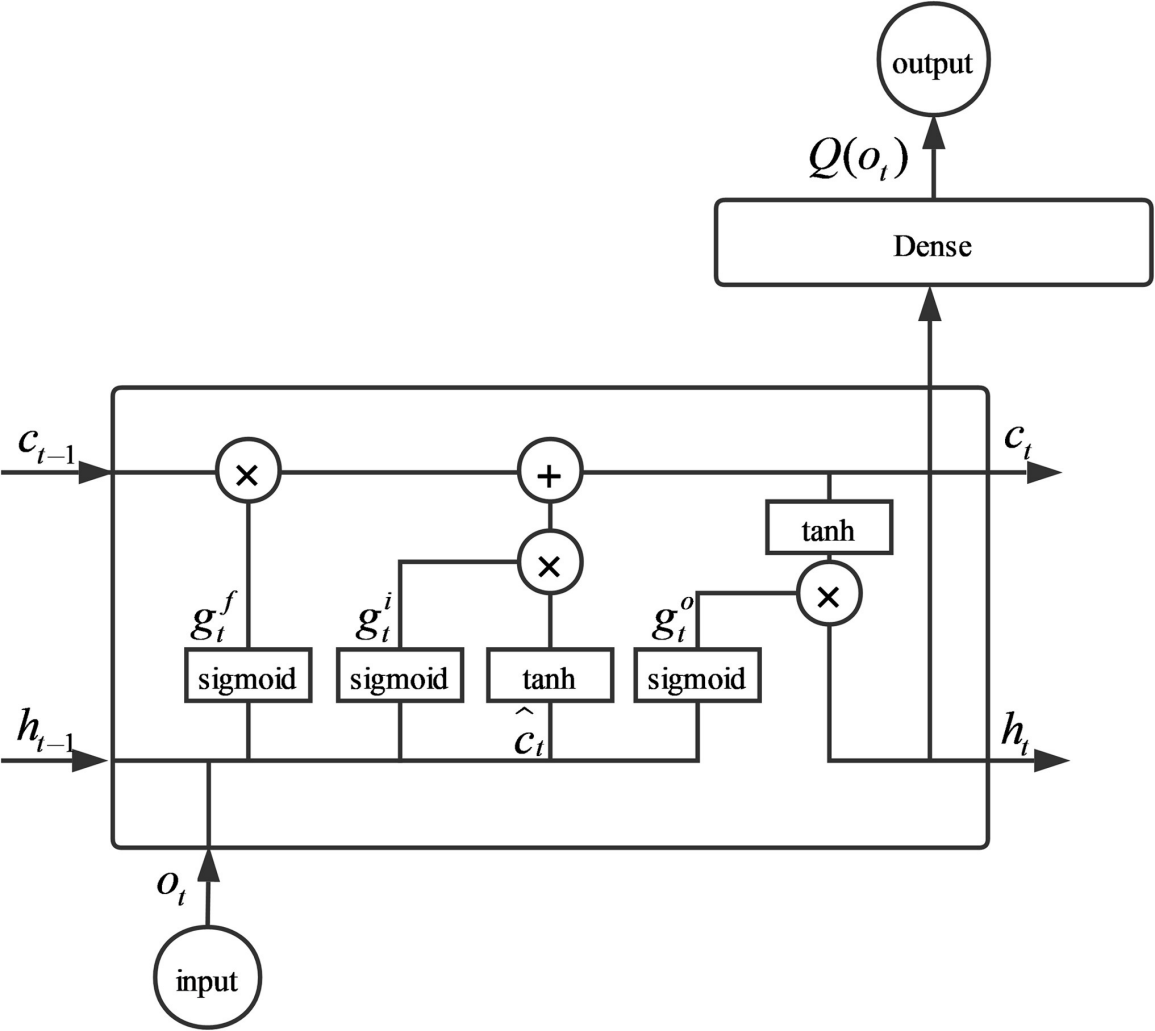
Model structure. A basic LSTM connected to a linear output layer is used here to implement the reinforcement one-shot active learning (ROAL) model that we proposed.

We use an LSTM network [43] connected to a linear output layer to implement the action-value function *Q*(*o*_*t*_, *a*_*t*_) in Q-learning, as shown in Fig 3. *Q*(*o*_*t*_) outputs a vector, in which each element corresponds to an action:

$$Q(o_{t},a_{t})=Q(o_{t})∙a_{t}$$ (8)

$$Q(o_{t})=W^{hq}h_{t}+b^{q}$$ (9)

Where *b*^*q*^ is the action-value bias, *h*_*t*_ is the output of the LSTM, *W*_*hq*_ represents the weights mapping from the LSTM output to the action-values. A basic LSTM is used in our model, and the equations are as follows:

$${\hat{g}}^{f},{\hat{g}}^{i},{\hat{g}}^{o},{\hat{c}}_{t}=W^{o}o_{t}+W^{h}h_{t-1}+b$$ (10)

$$g^{f}=\sigma ({\hat{g}}^{f})$$ (11)

$$g^{i}=\sigma ({\hat{g}}^{i})$$ (12)

$$g^{o}=\sigma ({\hat{g}}^{o})$$ (13)

$$c_{t}=g^{f}⨀c_{t-1}+g^{i}⨀tanh({\hat{c}}_{t})$$ (14)

$$h_{t}=g^{o}⨀tanh(c_{t})$$ (15)

Here, ${\hat{g}}^{f},{\hat{g}}^{i},{\hat{g}}^{o}$ respectively represent the forget gates, input gates and output gates. Where ${\hat{c}}_{t}$ is the candidate cell state and *c*_*t*_ represents the new LSTM cell state. *W*^*o*^ and *W*^*h*^ respectively represent the weights mapping from the observation to the gates and candidate cell state and the weights mapping from the hidden state to the gates and candidate cell state. *b* is the bias vector. σ(·) is a sigmoid function. ⨀ represents element-wise multiplication, and tanh(·) represents the hyperbolic tangent function.

## Experiments

We examined our proposed ROAL model under an AOL set-up for two image classification tasks and compared the experimental results of present study with the results from previous study. Our goal is to further study the following points through experiments: 1) whether the model we proposed can learn a practical strategy that knows how to label instances and when to instead request a label, and 2) whether the model effectively uses its uncertainty of instances to make decisions.

### Omniglot

#### Setup

We performed our first experiments on the Omniglot dataset [19], consisting of 1623 classes of characters from 50 different alphabets each hand-written by 20 different persons, for a total of 32460 instances. Following Woodward et al [23], we randomly divided the dataset into 1200 characters for training and kept the remaining 423 characters for testing. Our model interacted with classes of characters that it did not encounter during training to measure its test performance. To reduce the computational time of our experiments, images were downscaled to 28×28 pixels, and the pixel values were normalized between 0.0 and 1.0.

In each episode, 30 Omniglot images were randomly selected from 3 randomly sampled classes, without replacement. Here, the number of samples from each class may not have been balanced. Each selected class in the episode was assigned to a random label which was represented by a slot in a one-hot vector of length 3, giving *y*_*t*_. In order to reduce the risk of overfitting, we performed data augmentation for each class in the episode by randomly rotating in all samples from that class in {0°, 90°, 180°, 270°}. An LSTM with 200 hidden units was used here. We optimized the parameters of our model using Adam with the default parameters [49]. A grid search was performed over the following parameters, and the parameters of the results reported in this article are listed as follows. During training process, epsilon greedy exploration with *ϵ* = 0.23 was used for action selection. The discount factor *γ* was set to 0.5. Unless otherwise stated, each training and testing step consisted of a batch of 50 episodes, and the reward values were set as: *R*_*cor*_ = +1, *R*_*inc*_ = −1, and *R*_*req*_ = −0.05. For every 1000 episodes, we calculated the average accuracy, request, and precision rate. Notably, in order to achieve a better convergence effect, the learning rate of the model needs to be adjusted according to the change of the reward values, and the initial learning rate was set to 0.001. The training was carried out on 100,000 episodes. After that, 200 testing steps were conducted for evaluation.

### Results and discussion

**Fig 4:**
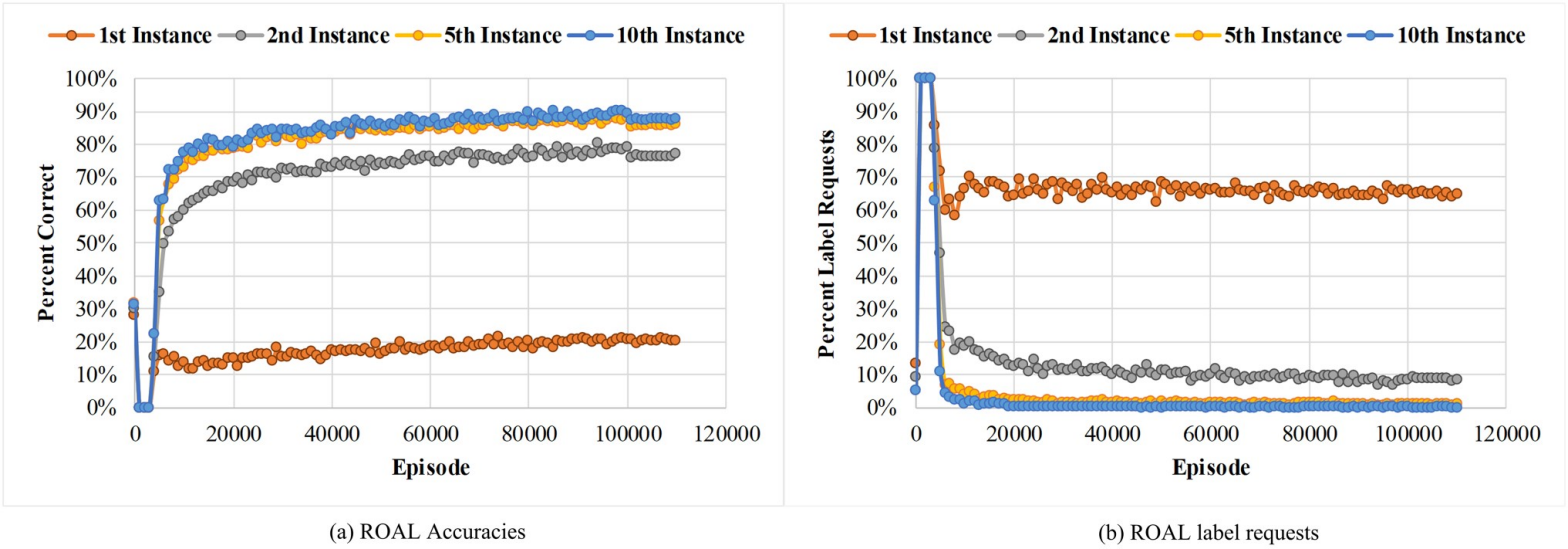
(a) ROAL Accuracies and (b) ROAL label requests per episode for the 1^st^, 2^nd^, 5^th^, and 10^th^ instances of all classes. The ROAL gains a higher accuracy while requests fewer labels on later instances of each class, indicating that the ROAL is performing “educated guesses” for new instances based on the instances it has already seen. At the 100,000 episode, the training stops and the data switches to test classes withheld from the training set.

**Fig 5:**
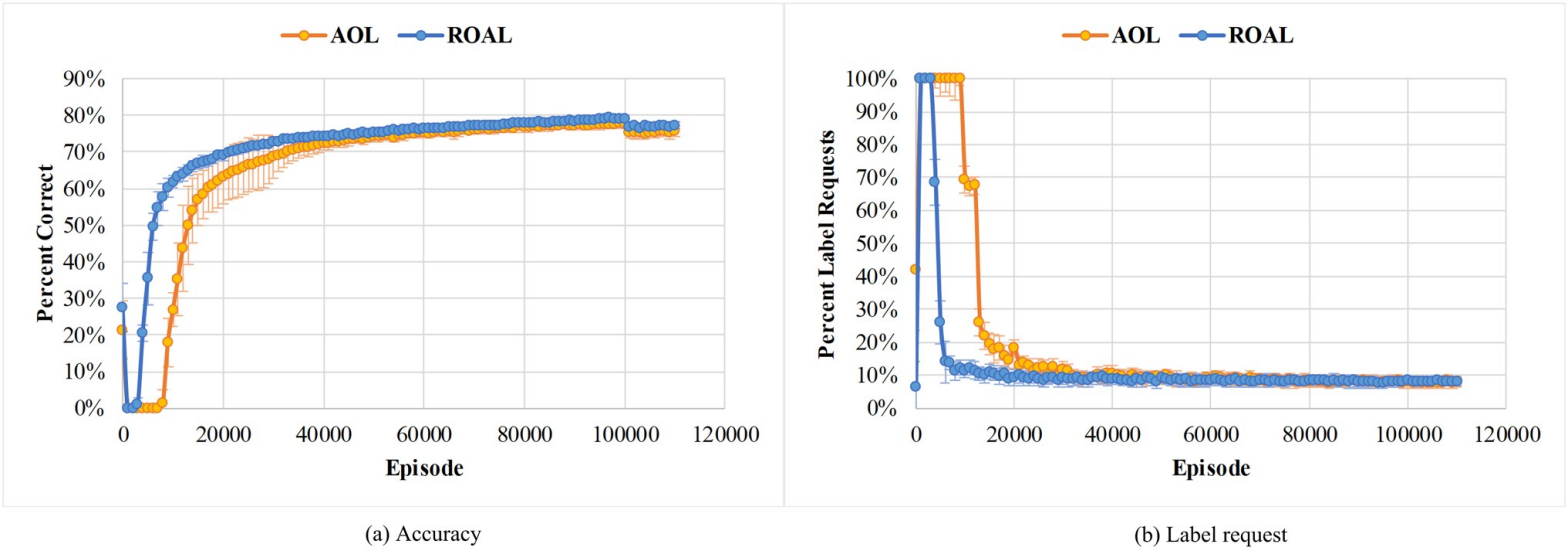
Comparison of overall (a) Accuracy and (b) Label request results between ROAL and AOL. Compared to AOL, ROAL is able to achieve higher accuracy and lower request rate in fewer iterations. After 100,000 episodes, the data switches to test set without further learning.

This section presents the results of the two experiments with our model. In the first experiment, we implemented both active one-shot learning (AOL) model with the default parameters from Ref. [23], and our ROAL model on the task in Fig 1. During training, the 1^st^, 2^nd^, 5^th^, and 10^th^ instances of all classes in each episode were identified. Notably, in this analysis, label requests were treated as incorrect label prediction when calculating the accuracy. After training on 100,000 episodes, the training is ceased. Then the model was given 10,000 more test episodes. In these episodes, no further update occurred, and the model was to run on previously unencountered classes pulled from a disjoint test set. We report the results in Figs 4 and 5.

As shown in Fig 4, first instance accuracy is poor, since the ROAL model that we propose learns to query the label for early instances of a class. We can also conclude that ROAL results in more predictions for later instances, since the label request rates of later instances decrease sharply. At the same time, the accuracy of the model is improved on later instances of a class, which approaches 90%. Fig 5 shows the average results of 10 repeated experiments. As shown in Fig 5, compared with AOL, ROAL has higher convergence speed, higher and more stable classification accuracy, and lower request rate. To evaluate the statistical significance of the comparison results on ROAL and AOL, Student’s paired two-tailed *t*-test was conducted. When the p-value in the hypothesis test was less than 0.05, the result was considered as significant. The statistical significance levels that accuracy and prediction are better in the case of ROAL than for AOL were substantially less than 0.05, suggesting that the results of ROAL are significantly superior to the results of AOL. These data indicate that ROAL greatly accelerates the training speed, and effectively avoids the phenomenon of low efficiency and the gradient vanishing problem in the early training stage, thus saving considerable time and computing resources by introducing cross entropy into the loss function.

**Fig 6:**
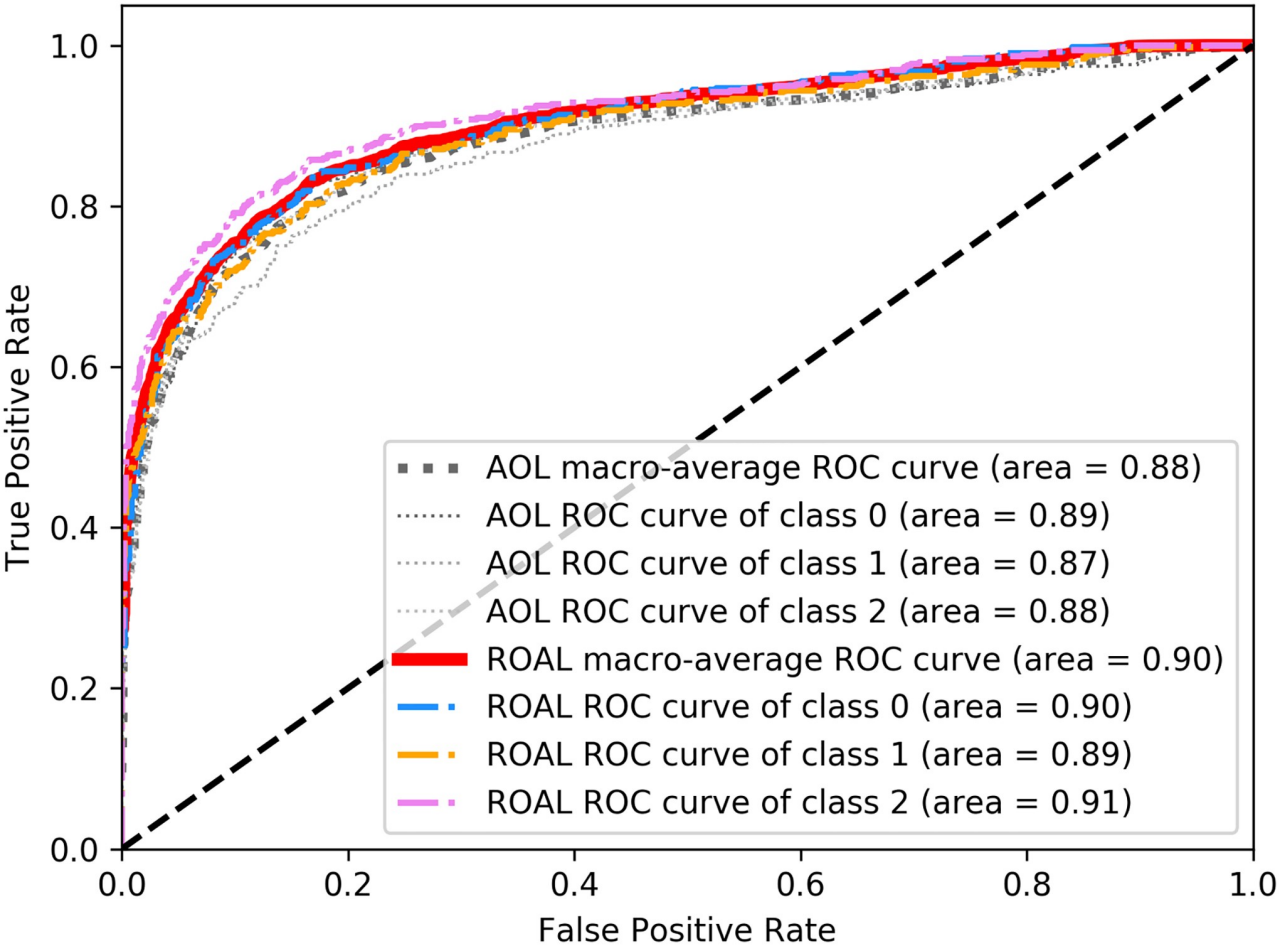
ROC plot with AUC values for AOL and ROAL.

To further compare the performance of the proposed ROAL method with the AOL method, Fig 6 shows the results of the receiver operating characteristic (ROC) curve analyses in our multiclassification task. The ROC curve, which is a plot of the true positive rate (TPR) against the false positive rate (FPR) at various threshold settings, can clearly illustrate the diagnostic ability of a classifier system. In a ROC plane, the axes range from 0 to 1, where FPR is plotted on the X-axis and the TPR is plotted on the Y-axis. The diagonal dotted straight line connecting (0,0) to (1,1) represents a random performance of the classifier. Any classifier that appears in the upper left triangle performs better than random guessing, while curves in the lower right of the ROC plot have worse classification performances. Since we are faced with the problem of multiclassification, we present not only the ROC curves of two algorithms for each class but also the macro-average ROC curves, reflecting the overall classification effect for both algorithms. As shown in Fig 6, the ROAL method had better upper-left ROC curve results than the AOL method.

The areas under the curve (AUC) of the ROC plot were also computed to quantitatively evaluate classification performance. The AUC can be calculated by using the trapezoidal areas created between each ROC point. The AUC value lies between 0 and 1, with a higher AUC value indicating better classification performance. As shown in Fig 6, the ROAL method had a higher macro-average AUC of 0.90 and higher AUC values for each class, while the AOL method had a macro-average AUC of 0.88. As a result, the ROC-AUC analyses show that the ROAL algorithm effectively improves the classification performance compared with the AOL algorithm.

In reinforcement learning, the setting of the reward function has a great influence on the convergence speed and the performance of the algorithm. To explore this, we further trained models using different reward values. Notably, when training the models of *R*_*inc*_ = −10 and *R*_*inc*_ = −20, for consistency of convergence, we used a batch size of 100. Our experimental setup was the same as Woodward’s. We used the default parameters from Woodward’s work to reproduce the results of AOL. At the same time, we show the best results we reproduced with the default parameters of the AOL model in Ref [23] presented on the same problem. Importantly, based on previous work, we further explored the impact of different *R*_*req*_ settings on the accuracy and request rate of the model. As shown in Table 1, our model obtains higher accuracy and a lower request rate with the same reward values setting. The experimental results also verified that the ROAL model can make trade-offs between high prediction accuracy with many label requests and few label requests but lower prediction accuracy. Higher prediction accuracy can be achieved by increasing the penalty value for wrongly predicting labels. Similarly, the request label rate can be reduced by increasing the penalty for the request label action, at the cost of accuracy. The results also indicate that if the reward value is set improperly, no label may be requested with random prediction or all the labels may be requested without any prediction. Therefore, proper setting of the reward value function has an important influence on the learning effect of the model.

**Table 1:** Test set classification accuracies and percentage of label requests per episode.

%	AOL				ROAL									
	Results in Ref [23]		*R*_*cor*_ = 1		*R*_*cor*_ = 1									
			*R*_*req*_ = −0.05		*R*_*req*_ = −0.05		*R*_*req*_ = −0.1		*R*_*req*_ = −1		*R*_*req*_ = −3		*R*_*req*_ = −4	
	Accuracy	Requests	Accuracy	Requests	Accuracy	Requests	Accuracy	Requests	Accuracy	Requests	Accuracy	Requests	Accuracy	Requests
Accuracy	75.9	7.2	75.3	7.9	78.8	7.9	**76.6**	**7.1**	33.7	0	33.2	0	33.8	0
Prediction	81.8	7.2	81.3	7.9	85.9	7.9	**82.5**	**7.1**	33.7	0	33.2	0	33.8	0
*R*_*inc*_ = −5 prediction	86.4	31.8	92.9	67.9	97	48.2	96.4	44.9	**91.5**	**20.6**	57	4.2	33.5	0
*R*_*inc*_ = −10 prediction	89.3	45.6	97.1	81.7	99.2	71.5	99.1	65.5	**97.8**	**42.9**	**91.1**	**16.2**	86.4	9.9
*R*_*inc*_ = −20 prediction	92.8	60.6	0	100	99.2	89.0	99.1	93.7	97.4	81.9	93.8	69.3	**92.8**	**52**

**Fig 7:**
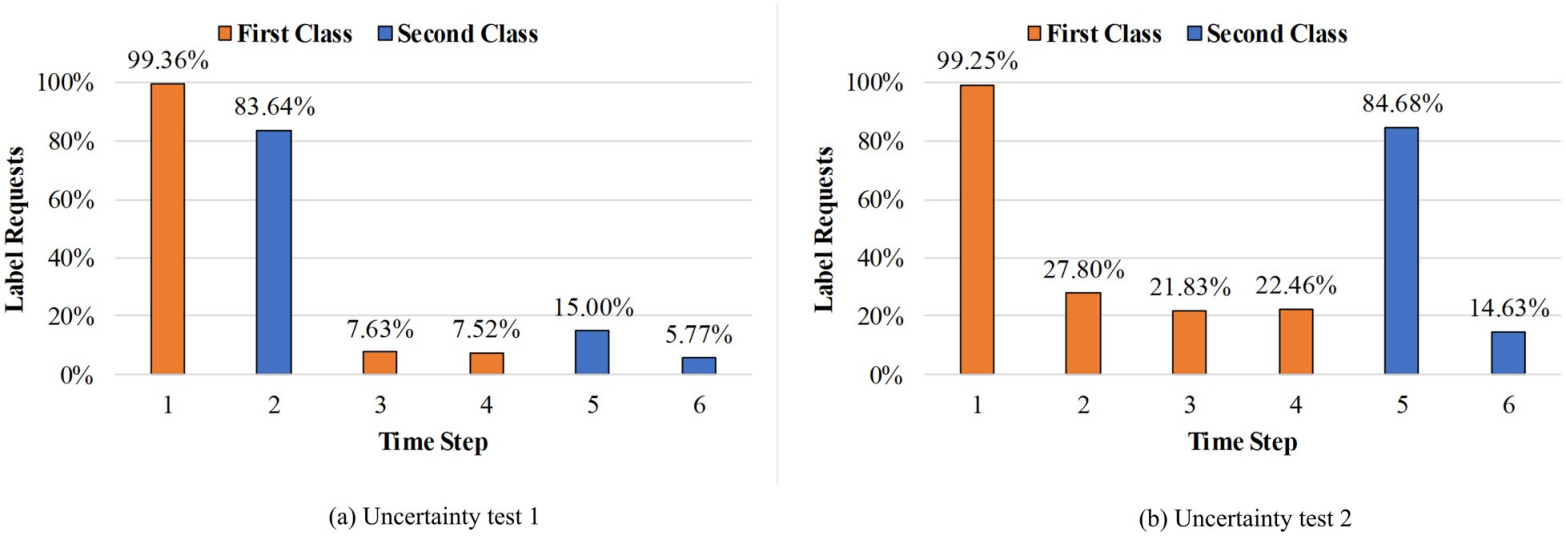
Second experiment results of the trained model. In this task, two random test classes were chosen for each episode. (a) At the beginning of each episode, we assigned two instances which came from different classes to the model. After that, two instances from each class was given, respectively. It shows that the request rate for later instances of the same class has been greatly reduced after the model saw an instance of that class. (b) 4 instances from the first class were presented, followed by 2 instances from the second class. The label request rate at time step 2 is greatly reduced, and the label request rate at time step 5 is greatly increased.

Finally, we performed another experiment to explore whether the model was effectively reasoning about its own uncertainty. In previous experiments, samples were randomly arranged in each episode. In this experiment, we artificially provided the order of the sample arrangement to explore the action strategy of model. In this task, experiments were carried out on the trained model, and two random test classes were selected for each episode. Our experiment was divided into two groups. In both groups, we ran 1000 episodes without learning and recorded the request percentage of episodes for each time step. In the first group, we assigned two instances that came from different classes to the mode at the beginning of each episode. Then, two instances from each class was given. As shown in Fig 7(a), the request rate for later instances of the same class was greatly reduced after the model saw an instance of that class. This result is consistent with the original intention of active learning. If representative samples can be effectively selected for labeling, then the cost of manual labeling can be greatly reduced. However, existing experiments still cannot prove whether the model selects actions based on uncertainty of instances or not, because it is likely to learn only a naive strategy that always requires labels in the first few steps. For further verification, we set the second group of experiments as: 4 instances from the first class were presented, followed by 2 instances from the second class. The results are shown in Fig 7(b). The label request rate at time step 2 was greatly reduced, and the label request rate at time step 5 was greatly increased. The difference in the request rate of these two time steps, and the similarity between the percentages of label requests of the both classes can finally show that the model selects an action based on the uncertainty of instances, because the model can increase the label request rate when a new class appears.

In Woodward’s paper [23], a supervised method in the same task was carried out. Compared with supervised learning with a label request rate is 100%, our model can achieve higher accuracy while using fewer labels at the same time.

### Handwritten alphanumeric characters

#### Setup

The second dataset included handwritten alphanumeric characters and consisted of 36 classes of characters, corresponding to digits from 0 to 9 and the letters from A to Z, with each class consisting of 39 instances. The input corresponds to 20×20 pixels image in binary format. We randomly divided the dataset into 28 characters for training, and kept the remaining 8 characters for testing.

Similar to the set-up of Omniglot, 30 images were randomly selected from several randomly sampled classes in each episode, without replacement. Data augmentation for each class in the episode was also performed. An LSTM with 200 hidden units was used here. Adam with the default parameters [49] was used here to optimize our model. A grid search was performed over the following parameters, and the parameters of the results reported in this article are listed as follows. Epsilon greedy exploration with *ϵ* = 0.4 was used. The discount factor *γ* was 0.6. The initial batch size was set to 50 and the reward values were set as: *R*_*cor*_ = +1, *R*_*inc*_ = −1, and *R*_*req*_ = −0.3. The initial learning rate was set to 0.002. The method of training and evaluation is the same as that for the Omnigolt data set.

### Results and discussion

In this section, we compare our ROAL model to AOL and a supervised learning model on handwritten alphanumeric characters recognition task. As introduced in Santoro et al. [25], the loss in the supervised learning model is the cross entropy between the true and predicted label, and the true label is always presented on the following time step. The same LSTM model was used in this supervised task for consistency, and the softmax modification is performed on the output without extra bits for the "request label" action. We expand the experiments by increasing the number of classes per episode. We report the results of prediction accuracy and request rate on the test sets in Table 2. For consistency of convergence, when training the models of 8 classes, a batch size of 100 was used, and the number of instances in each episode was changed to 80 in all three models.

**Table 2:** Results for ROAL and baselines for the handwritten alphanumeric characters classification.

%	3 classes		5 classes		8 classes	
	Accuracy	Requests	Accuracy	Requests	Accuracy	Requests
Supervised	89.1	100	78.9	100	76.2	100
AOL prediction	86.78	8.02	78.05	14.35*	72.23	11.06*
ROAL prediction	89.58*	6.8*	**79.17**	15.15	79.05*	15.36

(Results with statistical significance at the 0.05 level with respect to the Student’s paired *t*-test are marked with *.)

According to Table 2, the ROAL model also exhibits better performance than the AOL model on the handwritten alphanumeric characters dataset. At the same time, compared to the supervised learning model, the ROAL model significantly reduces the number of requests for tags while achieving the same or even higher accuracy. By increasing the number of classes per episode, we further demonstrate the ability of the ROAL algorithm to handle more complex tasks. We may conclude that the ROAL model has broad application prospects.

## Conclusions

We introduced a model that learns active learning via reinforcement learning. We evaluated the model on one-shot learning tasks. The results show that our model can transform from an engineering heuristic selection of samples to learning strategies from data. Compared to previous works [23], we substantially accelerated the convergence speed, avoided the gradient vanishing problem, improved the stability, reduced the number of request labels, and improved the accuracy of the model. The proposed model may be a good solution to practical problems such as movie recommendation [50] and network traffic analysis [20] due to its ability to learn and generalize new concepts in a short time.

In future work, we plan to evaluate our model on practical problems. For this, we may need a more sophisticated learning approach. Due to time and resources limitations, the parameters of our experiment may not be optimal; they can be optimized further to improve the performance of the algorithm.

## Supporting information

S1 Table Statistical test results of test episodes on Omniglot dataset (3 classes with *R*_*cor*_ = +1, *R*_*inc*_ = −1, and *R*_*req*_ = −0.05). (DOCX)

S2 Table Statistical test results of test episodes on handwritten alphanumeric characters dataset. (DOCX)
