# Supplementary material for: On the improvement of reinforcement active learning with the involvement of cross entropy to address one-shot learning problem
Source: PLoS One. 2019 Jun 19;14(6):e0217408. doi: 10.1371/journal.pone.0217408 (PMC6583946; doi:10.1371/journal.pone.0217408)
Supplement: S1 Table — (DOCX) [file pone.0217408.s001.docx]

S1 Table. Statistical test results of test episodes on Omniglot dataset (3 classes with $\boldsymbol{R}_{\boldsymbol{cor}}\mathbf{=+1,}\boldsymbol{R}_{\boldsymbol{inc}}\mathbf{=-1,}$ and $\boldsymbol{R}_{\boldsymbol{req}}\mathbf{=-0.05}$).

|  |  | AOL | ROAL |
| --- | --- | --- | --- |
| Accuracy | mean | 0.753 | 0.788 |
|  | std | 0.015 | 0.008 |
|  | median | 0.754 | 0.788 |
|  | p-value | 3.03E-09 | |
| Prediction | mean | 0.813 | 0.859 |
|  | std | 0.014 | 0.008 |
|  | medians | 0.815 | 0.860 |
|  | p-value | 2.09E-09 | |
| Request | mean | 0.079 | 0.079 |
|  | std | 0.005 | 0.003 |
|  | median | 0.080 | 0.080 |
|  | p-value | 0.595 | |
