# Supplementary material for: On the improvement of reinforcement active learning with the involvement of cross entropy to address one-shot learning problem
Source: PLoS One. 2019 Jun 19;14(6):e0217408. doi: 10.1371/journal.pone.0217408 (PMC6583946; doi:10.1371/journal.pone.0217408)
Supplement: S2 Table — (DOCX) [file pone.0217408.s002.docx]

S2 Table. Statistical test results of test episodes on handwritten alphanumeric characters dataset.

|  | |  | Supervised | AOL | ROAL |
| --- | --- | --- | --- | --- | --- |
| 3 classes | Prediction  Accuracy | mean | 0.8910 | 0.8678 | 0.8958 |
|  |  | median | 0.8914 | 0.8682 | 0.8957 |
|  |  | std | 0.0055 | 0.0072 | 0.0060 |
|  |  | p-value (-ROAL) | 0.0042 | 1.35E-07 |  |
|  | Requests | mean | 1.0000 | 0.0802 | 0.0680 |
|  |  | median | 1.0000 | 0.0801 | 0.0681 |
|  |  | std | 0.0000 | 0.0013 | 0.0003 |
|  |  | p-value (-ROAL) | 1.06E-51 | 1.56E-09 |  |
| 5 classes | Prediction  Accuracy | mean | 0.7890 | 0.7805 | 0.7917 |
|  |  | median | 0.7893 | 0.7807 | 0.7920 |
|  |  | std | 0.0057 | 0.0094 | 0.0063 |
|  |  | p-value (-ROAL) | 0.2035 | 0.0003 |  |
|  | Requests | mean | 1.0000 | 0.1435 | 0.1515 |
|  |  | median | 1.0000 | 0.1435 | 0.1515 |
|  |  | std | 0.0000 | 0.0016 | 0.0022 |
|  |  | p-value (-AOL) | 2.62E-43 |  | 3.65E-06 |
| 8 classes | Prediction  Accuracy | mean | 0.7620 | 0.7223 | 0.7905 |
|  |  | median | 0.7621 | 0.7220 | 0.7907 |
|  |  | std | 0.0037 | 0.0060 | 0.0060 |
|  |  | p-value (-ROAL) | 8.23E-10 | 1.66E-11 |  |
|  | Requests | mean | 1.0000 | 0.1106 | 0.1536 |
|  |  | median | 1.0000 | 0.1106 | 0.1537 |
|  |  | std | 0 | 0.0012 | 0.0016 |
|  |  | p-value (-AOL) | 1.55E-44 |  | 1.18E-12 |
